# ABCD^2^, ABCD^2^-I, and OTTAWA scores for stroke risk assessment: a direct retrospective comparison

**DOI:** 10.1007/s11739-022-03074-x

**Published:** 2022-08-20

**Authors:** Michele Domenico Spampinato, Marcello Covino, Angelina Passaro, Matteo Guarino, Beatrice Marziani, Caterina Ghirardi, Adelina Ricciardelli, Irma Sofia Fabbri, Andrea Strada, Antonio Gasbarrini, Francesco Franceschi, Roberto De Giorgio

**Affiliations:** 1 Department of Translational Medicine, University of Ferrara, Ferrara, Italy; 2 School of Emergency Medicine, University of Ferrara, Ferrara, Italy; 3 Emergency Medicine, Fondazione Policlinico Universitario Agostino Gemelli IRCCS, Università Cattolica del Sacro Cuore, Largo A. Gemelli, 8, 00168 Rome, Italy; 4 Emergency Medicine, Azienda Unità Sanitaria Locale, Ferrara, Italy; 5 Emergency Medicine, St. Anna Hospital, Ferrara, Italy; 6 Internal Medicine, Fondazione Policlinico Universitario Agostino Gemelli IRCCS, Università Cattolica del Sacro Cuore, Rome, Italy

**Keywords:** Acute stroke, Clinical predictive score, Emergency medicine, Transient ischemic attack

## Abstract

Transient ischemic attack (TIA) is a neurologic emergency characterized by cerebral ischemia eliciting a temporary focal neurological deficit. Many clinical prediction scores have been proposed to assess the risk of stroke after TIA; however, studies on their clinical validity and comparisons among them are scarce. The objective is to compare the accuracy of ABCD^2^, ABCD^2^-I, and OTTAWA scores in the prediction of a stroke at 7, 90 days, and 1 year in patients presenting with TIA. Single-centre, retrospective study including patients with TIA admitted to the Emergency Department of our third-level, University Hospital, between 2018 and 2019. Five hundred three patients were included. Thirty-nine (7.7%) had a stroke within 1 year from the TIA: 9 (1.7%) and 24 (4.7%) within 7 and 90 days, respectively. ABCD^2^, ABCD^2^-I, and OTTAWA scores were significantly higher in patients who developed a stroke. AUROCs ranged from 0.66 to 0.75, without statistically significant differences at each time-point. Considering the best cut-off of each score, only ABCD^2^ > 3 showed a sensitivity of 100% only in the prediction of stroke within 7 days. Among clinical items of each score, duration of symptoms, previous TIA, hemiparesis, speech disturbance, gait disturbance, previous cerebral ischemic lesions, and known carotid artery disease were independent predictors of stroke. Clinical scores have moderate prognostic accuracy for stroke after TIA. Considering the independent predictors for stroke, our study indicates the need to continue research and prompts the development of new tools on predictive scores for TIA.

## Introduction

Transient ischemic attack (TIA) is a temporary episode of neurologic dysfunction caused by focal brain, spinal cord, or retinal ischemia, without acute infarction or tissue injury [1]. Ischemia results from a critical reduction of cerebral blood flow due to local (e.g., atherosclerosis, inflammation, amyloid deposition, and arterial dissection) or systemic (e.g., cardiac embolism) mechanisms. Symptoms usually last less than an hour, suggesting a short-lived dysfunction of an area of the central nervous system. TIA is a common neurologic disorder [2] with a reported overall prevalence of 2% and an estimated incidence of 240,000 TIAs per year and an average annual risk for a subsequent ischemic stroke of 3–4% in the United States [3, 4], with incidence varying with age, reported equal to 0.52–2.37 and 0.05–1.14 in men and women aged 55–64, 0.94–3.39 and 0.71–1.47 in those aged 65–74, and 3.04–7.20 and 2.18–6.06 in those aged 75–84, respectively in the European population [5]. The diagnosis is established by clinical features and neuroimaging findings [6]. Since 2009, the American Heart Association (AHA) has replaced the classic “time-based” definition of TIA, centred on the short duration of symptoms, with the “tissue-based” definition, highlighting that even the short duration of symptoms can be due to permanent brain damage and that the use of neuroradiology tests is a fundamental step in the diagnostic process [1]. Conversely, The European Stroke Organization (ESO) defines TIA “as transient neurological symptoms, likely to be due to focal cerebral or ocular ischemia, which last less than 24 h” [7], resulting in high heterogeneity in the literature among published studies on TIA. However, a key aspect of the diagnosis of TIA is attributing symptoms to cerebral ischemia despite the absence of neuroradiological findings. Although clinical features may be non-specific, ischemic insult is the most likely cause when the attack is consistent with TIA being characterized by focal neurologic symptoms attributable to a single vascular territory. Transient ischemic attack can be considered a serious warning for an imminent ischemic stroke, with the highest risk in the first 48 h. Physicians should identify high-risk TIA patients and establish how quickly they should receive specialist assessment, brain-neurovascular imaging, and cardiac evaluation. Methods that can reliably assess the risk of stroke after TIA would be useful for triaging patients and guide the timing and setting of diagnostic/therapeutic strategies. The age, blood pressure, clinical features, duration of symptoms, and diabetes (ABCD^2^) is one of the most used assessment scores being an easy tool applied to identify patients at high risk of ischemic stroke in the first 7 days after TIA [8]. However, over the years, the ABCD^2^ predictive performance has been questioned as this score failed to reliably distinguish low- from high-risk subsets of patients with TIA [9]. Moreover, the predictive power of the ABCD^2^ score is generally lower in in-hospital patients compared to population-based settings, thus hampering the validity of this test in high-risk populations [10]. Furthermore, ABCD^2^ is based on the “time-based” definition of TIA [9]. Indeed, there is now evidence that findings indicative of acute ischemic lesions at diffusion-weighted magnetic resonance imaging (DWI-MRI) or acute or chronic ischemic lesions at computed tomography (CT) scan after a transient ischemic event are important predictors of stroke [11–13]. Risk models that combine information from acute DWI-MRI, non-invasive angiography, and presumed TIA aetiology could improve the accuracy of stroke risk prediction after TIA. In addition to ABCD^2^, many other risk stratification scores have been developed for TIA/stroke, i.e., the ABCD^2^-I that includes the ABCD^2^ items along with information about brain infarction detected at DWI-MRI or CT [13], and the recently published OTTAWA score that considers brain imaging, clinical features, and laboratory findings [14]. The ABCD^3^-I has been demonstrated to be superior to the ABCD^2^ and ABCD^2^-I scores [15, 16]. However, the ABCD3-I requires the inclusion of the results of the DWI-MRI, an imaging test not commonly available for evaluating patients with TIA in most EDs. Current European [7] and American [17] guidelines on TIA and stroke management, however, do not support the use of any clinical risk prognostic scores in the initial triage due to the lack of robust evidence on their use and the scarcity of recent studies comparing them. However, clinical prediction rules are extremely useful in the management of patients based on individual risk and are widely used in clinical practice. This study aimed to compare the prognostic accuracy of the ABCD^2^, ABCD^2^-I, and OTTAWA scores in the prediction of stroke within 7 and 90 days as well as 1 year in patients presenting with TIA in the Emergency Department (ED). Secondary outcomes are the evaluation of clinical characteristics, duration of symptoms, and the therapy used as prognostic factors.

## Methods

This is a retrospective, single-centre, 2-year cohort study. Our institutional electronic database was interrogated to enlist all patients aged > 18 years admitted to the ED of Arcispedale St. Anna, a referral centre for stroke in the Ferrara district, Cona, Ferrara, Italy, from January 1st, 2018, to December 31st, 2019, for “acute neurologic defect” or “TIA”. Only patients with a final diagnosis of TIA were included. According to our hospital protocol, all TIA diagnoses were established by neurological consultancy, using the “tissue-based” definition. All patients classified as TIA had no neurologic symptoms at the ED presentation, reported symptoms lasting < 24 h, and underwent a brain CT scan to exclude acute ischemic or haemorrhagic lesions. Patients with no neurologic symptoms and a new ischemic lesion on neuroimaging compatible with symptoms reported were defined as a “minor stroke” and excluded from the study. Clinical data were retrospectively extracted from our institutional electronic database including demographics, presenting, and accompanying symptoms, medical history, vital signs (blood pressure, heart rate, and peripheral blood oxygen saturation [SpO2]), and those specified by the ABCD^2^, ABCD^2^-I, and OTTAWA scores. The ABCD^2^ score, ABCD^2^-I, and OTTAWA scores were calculated in each patient according to the original studies [7, 12, 13] (see Table 1) on ED admission. The occurrence of stroke was defined by the re-admission to the ED with a new neurological defect and the presence of an ischemic lesion at neuroimaging, with the final diagnosis confirmed by a neurologist. Time points (i.e., 7 days, 90 days, and 1 year) were calculated from the date of the TIA leading to the first ED presentation.

**Table 1:** Characteristics of the clinical prediction scores

Clinical finding	Points
ABCD^2^ Score	
Age > 60 years	1 point
SBP > 140 mmHg or DBP > 90 mmHg,	1 point
Clinical symptom	Hemiparesis, 2 points Speech disturbances, 1 point Other, 0 point
Duration of symptoms	> 60 min, 2 points 10–59 min, 1 point < 10 min, 0 point
Diabetes mellitus	1 point
ABCD^2^-I Score	
Age > 60 years	1 point
SBP > 140 mmHg or DBP > 90 mmHg	1 point
Clinical symptom:	Hemiparesis, 2 points Speech disturbance, 1 point Other, 0 point
Duration of symptoms	> 60 min, 2 points 10–59 min, 1 point, < 10 min, 0 point
Diabetes mellitus	1 point
Any ischemic lesion at CT or MRI	3 points
OTTAWA Score	
The first episode of TIA	2 points
Duration of symptoms > 10 min	2 points
Known Carotid artery disease	2 points
Chronic therapy with an anti-platelets agent	3 points
Gait disturbance	1 point
Hemiparesis	1 point
Vertigo	− 3 points
DBP > 110 mmHg	3 points
Dysarthria	1 point
Atrial fibrillation	2 points
New or previous ischemic lesion on head CT or MRI	1 point
Platelet count > 400 × 10^3^/μL	2 points
Glycemia > 270 mg/dL	3 points

*CT* computed tomography, *DBP* diastolic blood pressure, *MRI* magnetic resonance imaging, *SBP* systolic blood pressure, *TIA* transient ischemic attack

Normally distributed data were described as mean and standard deviation (SD); not normally distributed data were described as the median and interquartile range (IQR); categorical data were reported as absolute numbers and percentages. Normally distributed data were compared via independent sample *t* test or Welch’s *t* test in case of unequal variance between groups. Not normally distributed data were compared via Mann–Whitney Test *U*. Pearson’s Chi-square test was used to compare categorical dependent variables among at least 2 independent groups.

The predictive ability of the scores was tested using the evaluation of the area under the receiver-operating characteristic (AUROC) curve. The AUROCs of the scores were compared using the method proposed by DeLong et al. [18]. The criterion associated with the highest Youden Index was considered the best cut-off. Multiple logistic regression analysis of each score (ABCD^2^; ABCD^2^;-I, and OTTAWA score) was performed for each outcome to evaluate the independent predictive power of each item.

Statistical analyses were performed using SPSS v.23 (Apache Software Foundation, Chicago, Illinois, USA) and MedCalc Version 17.6 (MedCalc Software BVBA). This study was approved by the Local Ethics Committee and conducted following the Helsinki Declaration.

## Results

**Fig. 1:**
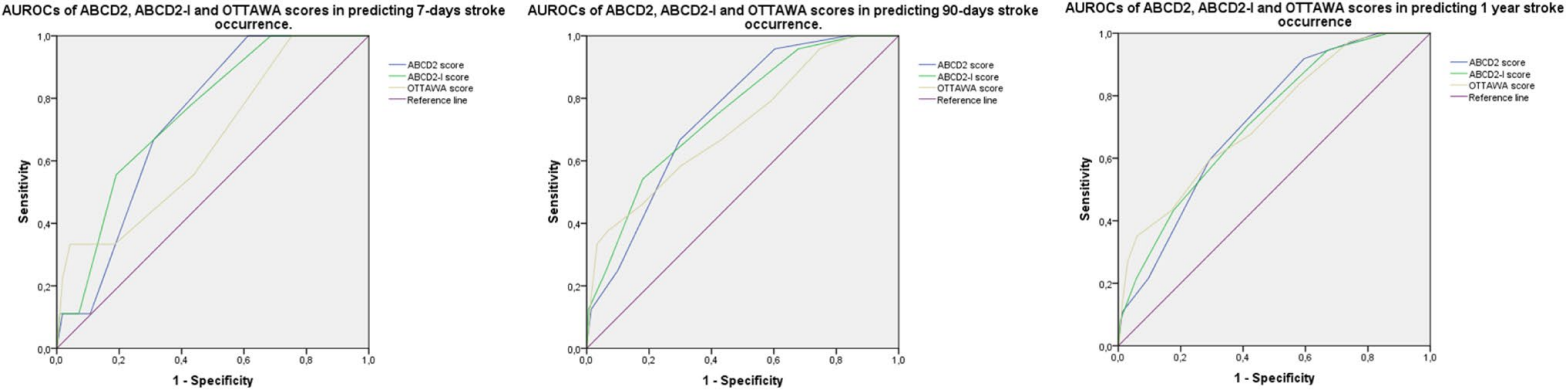
AUROCs of investigated scores at each time-point (7, 90 days and 1 year). For stroke within 7 days, ABCD^2^, ABCD^2^-I, and OTTAWA scores had an AUROC 0.73 (95% CI 0.61–083), 0.75 (95% CI 0.61–0.88), and 0.66 (95% CI 0.48–0.83), respectively; for stroke within 90 days, an AUROC of 0.75 (95% CI 0.71–0.78), 0.74 (95% CI 0.71–0.87), and 0.71 (95% CI 0.67–0.75), respectively; for stroke within 1 year, an AUROC of 0.69 (95% CI 0.65–0.73), 0.69 (95% CI 0.64–0.72), and 0.70 (95% CI 0.66–0.74), respectively

A total of 650 patients were initially included; of these, 147 patients were excluded, being classified as “acute neurologic deficit” but non-confirmed as a TIA by the neurologist. Finally, 503 patients were included in the study, 259 (51.5% male), with a median age of 77 years (IQR 25–75% 63–83 years). No patient had a haemorrhagic stroke or died during the year of observation. We found that 1.7% of patients developed a stroke within 7 days, 4.7% within 90 days, and 7.7% at 1 year from admission to the ED for TIA. As shown in Table 2, patients with hypercholesterolemia, previous TIA or stroke, carotid artery disease, duration of symptoms > 10 min and > 60 min, hemiparesis, and gait impairment were at higher risk of stroke. Conversely, patients with a previous or new chronic therapy with a statin, a beta-blocker, or low-weight molecular heparin and patients with a duration of symptoms < 10 min developed a significantly lower number of subsequent strokes. No differences were noted in the percentage of admitted patients between TIA patients who no developed stroke and patients who developed a stroke at each time-point. Clinical prediction scores were significantly higher in patients who developed stroke, while no differences were noted in diagnostic accuracy for the different outcomes, with AUROCs between 0.66 and 0.75. Evaluating the cut-offs of each score for each clinical outcome, only ABCD^2^ (with a score > 3) showed a sensitivity of 100% for stroke within 7 days, with an NPV of 100% (95% CI 97–100%) (Table 3 and Fig. 1). The multivariate analysis of each score showed the following results: for the ABCD^2^, “Hemiparesis” was the only independent predictor of stroke at each time-point, whereas both “Duration 10–59 min” and “Duration > 60 min” were independently predictive of stroke occurrence within 1 year. Among the item of the ABCD^2^-I, “Hemiparesis” was an independent predictor of stroke at each time-point, “Duration 10–59 min” and “Duration > 60 min” were independently predictive of stroke occurrence within 1 year, while “Ischemic lesion on head CT or MRI” was an independent predictor only with respect to stroke occurrence within 90 days. Finally, for the OTTAWA score, “Hemiparesis” was the only predictor of stroke within 7 days, whereas “Hemiparesis” and “Gait disturbance” were predictors of stroke within 90 days, and “Hemiparesis”, “Gait disturbance”, “First episode of TIA”, “ > 10-min symptom duration”, and “Known carotid artery disease” were independent predictors of stroke at 1 year (Table 4).

**Table 2:** Demographic and clinical data of the included patients

	Total, *N* = 503	No stroke,*N* = 464	Stroke within 7 days, *N* = 9 (1.7%)	*P* value	Stroke within 90 days, *N* = 24 (4.7%)	*P* value	Stroke within 1 year, *N* = 39 (7.7%)		*P* value
Age, median in years (IQR 25–75)	77 (63–83)	77 (64–83)	62 (52–72)	0.22	74 (59–81)	0.98	78 (63–84)	0.528	
Men, *N* (%)	259 (51.5)	240 (51.7)	7 (77.8)	0.11	12 (50)	0.88	19 (48.7)	0.718	
Hypertension, *N* (%)	333 (66.2)	302 (65.1)	6 (66.7)	0.97	18 (75)	0.35	31 (79.5)	0.068	
Hypercholesterolemia, *N* (%)	169 (33.6)	146 (31.5)	4 (44.4)	0.48	12 (50)	0.08	23 (59)	** < *0.001***	
Smoker, *N* (%)	51 (10.1)	46 (9.9)	1 (11.1)	0.92	2 (8.3)	0.76	5 (12.8)	0.52	
DM II, *N* (%)	103 (20.5)	91 (19.6)	2 (22.2)	0.89	5 (20.8)	0.96	12 (38.8)	0.097	
Obesity, *N* (%)	26 (5.2)	24 (5.2)	1 (11.1)	0.41	2 (8.3)	0.47	2 (5.1)	0.9	
CIC, *N* (%)	122 (24.3)	109 (23.5)	3 (33.3)	0.52	8 (33.3)	0.28	13 (33.3)	0.168	
Previous stroke or TIA, *N* (%)	90 (17.9)	75 (16.2)	1 (11.1)	0.59	9 (37.5)	***0.01***	15 (38.5)	** < *0.001***	
PAD, N (%)	15 (3)	13 (2.8)	1 (11.1)	0.26	2 (8.3)	0.11	2 (5.1)	0.414	
Previous chronic therapy with:									
ASA, *N* (%)	172 (34.2)	160 (34.5)	4 (44.4)	0.51	7 (29.2)	0.59	12 (30.8)	0.639	
Other anti-platelet agents, *N* (%)	54 (10.7)	48 (10.3)	0 (0)	0.29	3 (12.5)	0.77	6 (15.4)	0.329	
Coumadin, *N* (%)	38 (7.6)	34 (7.3)	0 (0)	0.38	3 (12.5)	0.34	4 (10.3)	0.506	
DOAC, *N* (%)	24 (4.8)	21 (4.5)	0 (0)	0.49	1 (4.2)	0.88	3 (7.7)	0.373	
Statin, *N* (%)	137 (27.2)	119 (25.6)	3 (33.3)	0.68	8 (33.3)	0.49	18 (46.2)	***0.006***	
ACE-I, *N* (%)	197 (39.2)	179 (38.6)	3 (33.3)	0.71	10 (41.7)	0.79	18 (46.2)	0.352	
Beta-blockers, *N* (%)	134 (26.6)	117 (25.2)	4 (44.4)	0.22	10 (41.7)	0.08	17 (43.6)	***0.013***	
Discharged with chronic therapy									
ASA, *N* (%)	241 (48.1)	219 (47.4)	7 (77.8)		15 (62.5)	0.14	22 (56.4)	0.28	
Clopidogrel, *N* (%)	140 (27.9)	129 (27.9)	2 (22.2)	0.51	6 (25)	0.74	11 (28.2)	0.97	
LWMH, *N* (%)	14 (2.8)	10 (2.2)	2 (22.2)	0.29	3 (12.5)	**0.003**	4 (10.3)	***0.003***	
Coumadin, *N* (%)	38 (7.6)	35 (7.6)	0 (0)	0.38	3 (12.5)	0.35	3 (7.7)	0.98	
DOAC, *N* (%)	25 (5)	21 (4.5)	0 (0)	0.49	1 (4.2)	0.84	4 (10.3)	0.116	
STATIN, *N* (%)	186 (37.1)	162 (35.1)	5 (55.6)	0.67	12 (50)	0.18	24 (61.5)	***0.001***	
ACE-I, *N* (%)	222 (44.3)	200 (43.3)	5 (55.6)	0.71	13 (54.2)	0.31	22 (56.4)	0.113	
Beta-blockers, *N* (%)	138 (27.5)	117 (25.3)	5 (55.6)	0.22	13 (54.2)	**0.003**	21 (53.8)	** < *0.001***	
SBP, median (IQR 25–75), mmHg	145 (130–160)	145 (130–160)	150 (140–160)	0.89	148 (140–160)	0.99	150 (135–160)	10.54	
DBP, median (IQR 25–75), mmHg	80 (70–90)	80 (70–90)	90 (80–100)	0.37	80 (8–90)	0.71	80 (80–90)	0.775	
HR, median (IQR 25–75), mmHg, ppm	75 (66–80)	75 (67–80)	65 (61–80)	0.49	70 (64–81)	0.88	75 (65–81)	0.86	
Creatinine, median (IQR 25–75), mg/dL	0.94 (0.78–1.14)	0.94 (0.78–1.14)	1.02 (0.97–1.27)	0.17	0.89 (0.8–1.03)	0.55	0.91 (0.79–1.08)	0.661	
Glicemia mg/d, median (IQR 25–75)	114 (101–139)	113 (101–140)	113 (108–131)	0.72	114 (109–127)	0.82	114 (106–129)	1	
Platelet count × 100/mmc, median (IQR 25–75)	222 (180–266)	223 (181–266)	209 (192–276)	1	221 (182–290)	1	221 (180–276)	0.611	
Admitted to the hospital from ED, *N* (%)	141 (28)	129 (27.1)	4 (44.4)	0.27	10 (41.7)	0.12	14 (37.8)	0.161	
ABCD^2^ score, median (IQR 25–75)	4 (3–5)	4 (3–5)	5 (4–5)	0.055	5 (4–5.5)	** < 0.001**	5 (4–5)	***0.001***	
ABCD^2^ score > best cut-off for each time-point, *N* (%)	–	303 (61.3)	9 (100)	**0.02**	10 (66.7)	0.01*	15 (78.9)	0.121^$^	
ABCD^2^ I score, median (IQR 25–75)	4 (3–5)	4 (3–5)	6 (5–6)	0.08	6 (4.5–6.5)	0.003*	5 (4–6)	0.004*	
ABCD^2^ I score, > best cut-off for each time-point, *N* (%)	–	94 (19)	5 (55)	**0.006**	8 (53.3)	0.002*	12 (63%)	***0.045***^***$***^	
OTTAWA score, median (IQR 25–75)	6 (5–8)	6 (4–8)	7 (6–11)	0.72	8 (6–11)	0.39	8 (6–11)	***0.014***	
OTTAWA score > best cut-off for each time-point, *N* (%)	–	21 (4.3)	3 (33.3)	** < *0.001***	60 (40)	< 0.001*	6 (36.8)	** < *0.001***^***$***^	

Bolditalic values indicate statistically significant *p* values (*p* < 0.05)

*ACE-I* angiotensin-converting-enzyme inhibitors, *ASA* acetylsalicylic acid, *CIC* chronic ischemic cardiomyopathy, *CT* computed tomography, *DBP*, diastolic blood pressure, *DM II* diabetes mellitus type 2, *DOAC* direct oral anti-coagulant, *HR* heart rate, *IQR* inter quartile range, *LWMH* low-weight molecular heparin, *MRI* magnetic resonance imaging, *N* number of cases, *PAD* peripheral artery disease, *SBP* systolic blood pressure

*P value referred to the comparison to the “no stroke between 90 days” group, not shown in the table

^$^*P* value referred to the comparison to the “no stroke between 1 year” group, not shown in the table

**Table 3:** Accuracy and characteristics of the investigated scores at each time points (7, 90 days and 1 year)

	AUROC (IC 95%)	Best cut-off	Sensitivity, % (95% IC)	Specificity, % (95% IC)	PPV, % (95% CI)	NPV % (95% CI)
7 day stroke occurrence						
ABCD^2^ Score	0.73 (0.61–083)	> 3	100 (62–100)	38.66 (34–43)	2.8 (1.4–5.5)	100 (97–100)
ABCD^2^-I Score	0.75 (0.61–0.88)	> 5	77 (40–96)	57 (52–61)	3 (1–6)	99 (97–99)
OTTAWA SCORE	0.66 (0.48–0.83)	> 10	33 (9–69)	95 (93–97)	12 (3–33)	98 (97–99)
90 day stroke occurrence						
ABCD^2^ Score	0.75 (0.71–0.78)	> 4	66.67 (40–87)	70.15 (65–73)	6.2 (3.2–11.5)	98.5 (96.4–9.4)
ABCD^2^-I Score	0.74 (0.71–0.87)	> 5	54 (33–73)	82 (78–85)	13 (7–21)	97 (95–98)
OTTAWA SCORE	0.71 (0.67–0.75)	> 9	37 (19–59)	93 (90–95)	21 (11–38)	96 (94–98)
1 year stroke occurrence						
ABCD^2^ score	0.69 (0.65–0.73)	> 3	91 (76–97)	40 (35–45)	10 (7–15)	98 (95–99)
ABCD^2^-I score	0.69 (0.64–0.72)	> 4	70 (52–83)	58 (54–63)	11 (8–17)	96 (92–97)
OTTAWA SCORE	0.70 (0.66–0.74)	> 9	35 (20–52)	93 (91–95)	31 (18–48)	94 (92–96)

*PPV* positive predictive value, *NPV* negative predictive value

**Table 4:** Multivariate analysis of the items of the ABCD^2^, ABCD^2^-I, and OTTAWA scores in the prediction of stroke within 7 and 90 days and at 1 year after TIA

	ABCD^2^ score		ABCD^2^-I score		OTTAWA score	
	OR (95% CI)	*P* value	OR (95% CI)	*P* value	OR (95% CI)	*P* value
Within 7 days						
Age > 60 years	0.1 (0.11–1.68)	0.22	0.22 (0.042–1.08)	0.065		
SBP > 140 or DBP > 90 mmhg	2.23 (0.43–11.4)	0.34	2.41 (0.45–12.88)	0.31		
Other	*Ref*		*Ref*			
Speech disturbance	0 (0–17.25)	0.99	0 to + ∞	0.99	5.01 (0.93–25.1)	0.06
Hemiparesis	8 (1–68)	***0.05***	7 (1–63.31)	***0.05***	30.45 (3.09–299.8)	***0.003***
Duration < 10 min	*Ref*		*Ref*			
Duration 10–59 min	0 to + ∞	0.99	0 to + ∞	0.99		
Duration > 60 min	0 to + ∞	0.99	0 to + ∞	0.99		
DM II	1.21 (0.26–6.49)	0.82	1.16 (0.21–6.49)	0.86		
Ischemic lesion at head CT or MRI			4.72 (0.96–23.07)	0.055	1.98 (0.41–9.46)	0.39
The first episode of TIA					0.82 (0.12–5.3)	0.82
Duration > 10 min					0 to + ∞	0.99
Known carotid artery disease					0.53 (0.07–3.95)	0.54
Chronic therapy with an anti-platelets agent					1.41 (0.29–6.89)	0.66
Gait disturbance					3.04 (0.59–15.24)	0.18
Vertigo					2.3 (0.19–27.4)	0.5
DBP 110 mmHg					4.6 (0.35–60.41)	0.24
Atrial fibrillation					0.73 (0.07–8.17)	0.8
Platelets count > 400 × 10^3^/μL					0 to + ∞	0.99
Glycemia > 270 mg/dL					0 to + ∞	0.99
Within 90 days						
Age > 60 years	0.93 (0.33–2.58)	0.89	0.63 (0.21–1.88)	0.63		
SBP > 140 or DBP > 90 mmhg	2.44 (0.86–6.93)	0.092	2.64 (0.917–7.64)	0.72		
Other	*Ref*		*Ref*			
Speech disturbance	0.85 (0.16–4.43)	0.85	0.87 (0.16–4.61)	0.87	1.36 (0.42–4.36)	0.59
Hemiparesis	6.79 (1.91–24.29)	***0.003***	6.54 (1.8–23.66)	***0.004***	7.43 (2.65–20.85)	** < *0.001***
Duration < 10 min	*Ref*		*Ref*			
Duration 10–59 min	0 to + ∞	0.99	0 to + ∞	0.99		
Duration > 60 min	0 to + ∞	0.99	0 to + ∞	0.99		
DM II	0.89 (0.31–2.78)	0.89	0.81 (0.27–2.42)	0.71		
Ischemic lesion at head CT or MRI			2.97 (1.16–7.59)	***0.023***	1.36 (0.42–4.28)	0.67
The first episode of TIA					3.33 (0.7–15.84)	0.13
Duration > 10 min					0 to + ∞	0.99
Known carotid artery disease					2.62 (0.93–7.39)	0.067
Chronic therapy with an anti-platelets agent					0.84 (0.26–2.73)	0.78
Gait disturbance					4.41 (1.47–13.14)	***0.008***
Vertigo					0.31 (0.02–3.53)	0.35
DBP 110 mmHg					0 to + ∞	0.99
Atrial fibrillation					2.63 (0.71–9.75)	0.15
Platelets count > 400 × 10^3^/μL					8.86 (0.7–112)	0.09
Glycemia > 270 mg/dL					4.82 (0.39–59.2)	0.21
Within 1 year						
Age > 60 years	1.44 (0.55–3.75)	0.44	1.19 (0.44–3.22)	0.71		
SBP > 140 or DBP > 90 mmhg	1.52 (0.7–3.3)	0.29	1.55 (0.71–3.39)	0.26		
Other	*Ref*		*Ref*			
Speech disturbance	0.93 (0.32–2.65)	0.89	0.92 (0.32–2.64)	0.88	1.07 (0.51–2.97)	0.84
Hemiparesis	3.02 (1.22–7.48)	***0.017***	2.87 (1.15–7.58)	***0.024***	2.79 (1.33–5.85)	***0.003***
Duration < 10 min	*Ref*		*Ref*			
Duration 10–59 min	10.13 (1.3–78.9)	***0.027***	10.01 (1.29–78)	***0.027***		
Duration > 60 min	14.57 (1.92–110.57)	***0.01***	14.19 (1.86–107.79)	***0.01***		
DM II	1.93 (0.9–4.14)	0.09	1.81 (0.83–3.91)	0.17		
Ischemic lesion at head CT or MRI			1.66 (0.8–3.43)	0.17	1.45 (0.53–3.97)	0.46
The first episode of TIA					3.19 (1.21–8.42)	***0.019***
Duration > 10 min					14.99 (1.97–113.99)	***0.009***
Known carotid artery disease					3.11 (1.41–6.89)	***0.005***
Chronic therapy with an anti-platelet agent					1.28 (0.61–2.71)	0.52
Gait disturbance					3.81 (1.58–9.81)	***0.003***
Vertigo					0.25 (0.05–1.36)	0.11
DBP 110 mmHg					0.56 (0.06–4.88)	0.59
Atrial fibrillation					2.39 (0.66–8.73)	0.18
Platelets count > 400 × 10^3^/μL					2.75 (0.29–26.52)	0.37
Glycemia > 270 mg/dL					1.26 (0.13–12.04)	0.83

Bolditalic values indicate statistically significant *p* values (*p* < 0.05)

*CT* computed tomography, *DBP* diastolic blood pressure, *DM II* diabetes mellitus type 2, *MRI* magnetic resonance imaging, *Ref* the item “Other” and “Duration < 10 min” of ABCD^2^ and ABCD^2^-I score was considered as reference categories for “Speech disturbance” and “Hemiparesis” and for “Duration 10–59 min” and “Duration > 60 min”, according to the original scores (see Table 1), *SBP* systolic blood pressure, *TIA* transient ischemic attack

## Discussion

Clinical scores provide a probability estimate of adverse events by assigning a specific score to some clinical and laboratory parameters [19]. Clinical scores were reported to be superior to isolated clinical judgment, because they collect the experience of many clinical cases and can objectively weigh the role of each item in the construction of the overall risk of a short-term adverse event [20, 21]. However, as demonstrated by Liao and Mark [20], physicians seem reluctant to use scores. One possible explanation is that there are many clinical prediction scores and identifying the best one in terms of ease of use and prognostic accuracy is often difficult. According to Chaudhary et al. [22], clinical scores developed for the prediction of stroke after a TIA are highly heterogeneous in terms of methodologies (i.e., different diagnostic criteria, e.g., “time-based” vs. “tissue-based”) and wide variability of the investigated patients (TIA or stroke or a combination of TIA and stroke patients). Also, Perry et al. [23] reported that the median sensitivity of clinical scores for TIA expected by physicians was higher than that reached by any existing scores, thus limiting their value in daily practice. An early diagnosis of TIA and a correct evaluation of several cardiovascular risk factors may aid adequate patient management leading to reduced rates of stroke, myocardial infarction, and vascular death as well as improved quality of life [24, 25]. According to our results, ABCD^2^, ABCD^2^-I, and OTTAWA risk scores have moderate diagnostic accuracy, with an AUROC < 0.75 in predicting the occurrence of stroke within 7 and 90 days, and at 1 year. In addition to the complexity of the OTTAWA score (which includes clinical, anamnestic, and laboratory data), there is no significant difference between this score and the ABCD^2^ and the ABCD^2^-I for all outcomes. As highlighted by the PROMAPA study [26], clinical scores were not able to replace a diagnostic evaluation, including blood tests, neuroradiologic and vascular imaging, and cardiac monitoring. Weimar et al. and Zhao et al. reported a low accuracy of clinical predictive scores for stroke [27, 28]. Specifically, Weimar et al. [27], who conducted a prospective cohort study in 16 German neurology departments, recruited 1897 consecutive patients with TIA or acute stroke and showed that all clinical predictive scores had an AUROC < 0.65 with low sensitivity and specificity. To assess the power of stroke prediction of ABCD^2^, Zhao et al. [28] performed a diagnostic meta-analysis and applied the results to a hypothetical cohort of 1000 patients with TIA. The pooled data of ABCD^2^ at 7 and 90 days showed a sensitivity of 79.9% and 76.6%, respectively, and a specificity of 29.2% and 40.3%, respectively. A recent paper by Perry et al. [29], including 7607 patients from 13 Canadian EDs, identified an AUROC for the OTTAWA TIA risk score of 0.70 (95% CI 0.66–0.73), which is a finding comparable to ours. However, while Perry et al. demonstrated that the OTTAWA score was significantly higher than ABCD^2^ (AUROC of 0.60; 95% CI 0.55–0.64) in predicting stroke at 1 week, our data revealed no significant difference among the investigated scores. Considering the best cut-off of each score, ABCD^2^ > 3 showed a sensitivity of 100% in the prediction of stroke within 7 days from the occurrence of TIA, with a negative predictive value (NPV) of 100%. This suggests that ABCD^2^ can be useful in excluding patients having a stroke in the short term (7 days). However, the limited number of patients enrolled in this study and the relatively low number of ischemic events prevent us to drawn firm conclusions on the NPV of ABCD^2^ in the short term. Since ABCD^2^-I and OTTAWA showed an NPV < 99% in the short, medium, and long term, these scores should not be used in excluding patients at risk of stroke occurrence.

Concerning the items of ABCD^2^ and ABCD^2^-I scores, only hemiparesis was an independent predictor of stroke, whereas hemiparesis and speech disturbance were independent predictors of stroke within 90 days. Regarding OTTAWA, hemiparesis predicted all outcomes, whereas the duration of symptoms and the known carotid artery disease were predictors of stroke within 1 year. Various authors have suggested adding the evaluation of brain or carotid imaging to the clinical scores to improve their diagnostic accuracy [30, 31]. However, in our series, the presence of any ischemic lesions at CT scan was an independent predictor of stroke in the subsequent 90 days and the presence of known carotid artery stenosis was a significant predictor within 90 days and at 1 year without increasing the accuracy of the ABCD^2^-I and OTTAWA vs. ABCD^2^. The multivariate analysis of each score at each time-point showed that despite the complexity of these scores, only a few elements appeared useful in identifying patients at higher risk of stroke. Moreover, as indicated in Table 2, cardiovascular risk factors and therapies have a potential role in the development of stroke at 90 days and 1 year. Thus, an ideal score that considers the right clinical elements, risk factors, and long-term therapies are expected to better predict the probability of a subsequent stroke.

Although this study is one of the few comparing the most applied clinical predictive scores for TIA in EDs, it has some limitations due to the retrospective nature of our database. First, the restricted access to the full set of patients’ information, including other imaging tests or exams performed, may have underestimated the total number of strokes in the 2-year investigational period. Second, despite the TIA protocol in our hospital being based on major guidelines for stroke, the decision to start a treatment or skip diagnostic investigations may have been taken on a case-by-case scenario, thus affecting the outcome. Third, the ABCD2-I original study [13] relies on the “time-based” TIA definition and assigned three points for a new ischemic lesion at brain DWI-MRI or any ischemic lesion at brain CT; however, patients with a new ischemic lesion compatible with the reported symptoms were considered as “minor stroke” and excluded from the study as potential confounders affecting the diagnostic accuracy of the score. Since no patients underwent DWI-MRI, a new ischemic lesion could remain undetected, thereby potentially affecting the “tissue-based” TIA definition used in our study. However, DWI-MRI is rarely performed in the EDs for TIA and all included patients also fulfilled the “time-based” definition of TIA. Finally, the limited number of patients with ischemic stroke in relation to the study endpoints (i.e., 7 days, 90 days, and 1 year) is likely to have downsized the statistical power of the study.

## Conclusions

In conclusion, clinical prediction scores may be useful in managing patients with TIA in the ED and help stratify patients according to individual risk of stroke; however, this work showed that clinical scores have only moderate prognostic accuracy for stroke after TIA, with no differences among them at any time-point. Considering the independent predictors for stroke, our study indicates the need to continue research and prompts the development of new tools on predictive scores for TIA.
